# Stress cardiomyopathy in vascular Ehlers-Danlos syndrome: first case report and proposed mechanisms

**DOI:** 10.1093/eschf/xvag100

**Published:** 2026-05-12

**Authors:** Xiaoru Che, Zhenzhen Wang, Xiaohua Tang, Chuanjing Zhang, Wei Wang, Qi Chen, Ying Zhou

**Affiliations:** Department of Cardiology, Zhejiang Provincial People’s Hospital, People’s Hospital of Hangzhou Medical College, Hangzhou, Zhejiang 310014, P.R. China; Department of Cardiology, Zhejiang Provincial People’s Hospital, People’s Hospital of Hangzhou Medical College, Hangzhou, Zhejiang 310014, P.R. China; Department of Cardiology, Zhejiang Provincial People’s Hospital, People’s Hospital of Hangzhou Medical College, Hangzhou, Zhejiang 310014, P.R. China; Department of Cardiology, Zhejiang Provincial People’s Hospital, People’s Hospital of Hangzhou Medical College, Hangzhou, Zhejiang 310014, P.R. China; Department of Cardiology, Zhejiang Provincial People’s Hospital, People’s Hospital of Hangzhou Medical College, Hangzhou, Zhejiang 310014, P.R. China; Department of Cardiology, Zhejiang Provincial People’s Hospital, People’s Hospital of Hangzhou Medical College, Hangzhou, Zhejiang 310014, P.R. China; Department of Cardiology, Zhejiang Provincial People’s Hospital, People’s Hospital of Hangzhou Medical College, Hangzhou, Zhejiang 310014, P.R. China

**Keywords:** Vascular Ehlers-Danlos syndrome, COL3A1, Stress cardiomyopathy, Takotsubo syndrome, Type III collagen, Myocardial interstitium

## Introduction

Vascular Ehlers-Danlos syndrome (vEDS) represents the most severe form of Ehlers-Danlos syndrome (EDS), caused by heterozygous mutations in the COL3A1 gene encoding type III collagen. Defective type III collagen production causes significant fragility in blood vessels and hollow organs, predisposing patients to spontaneous arterial dissections, ruptures, and intestinal perforations.^1^

While cardiac complications in EDS patients have been sporadically reported, including cardiomyopathy hypertrophic obstructive and non-compaction cardiomyopathy,^2,3^ the association between vEDS and stress cardiomyopathy (Takotsubo syndrome) has not been previously described in the literature. We report the first case of genetically confirmed vEDS presenting with stress cardiomyopathy, exploring the potential molecular mechanisms underlying this novel association and its clinical implications for vEDS management.

## Case presentation

A 36-year-old male presented to a local hospital on 25 August 2025, with abdominal pain. Initial abdominal CT showed left anterior renal fascia thickening with fat stranding. Conservative management was initiated with fasting, fluid resuscitation, and antibiotics.

Two days later, the patient developed worsening symptoms and hypotension. Emergency CT angiogram revealed haemoperitoneum from a suspected ruptured left hepatic artery aneurysm, bilateral renal artery dissections with infarctions, and a left iliac artery dissection. He underwent emergency laparotomy and left lateral hepatectomy, with an estimated blood loss of 3000 mL, requiring massive transfusion The patient was then transferred to the intensive care unit for postoperative care.

After stabilization, the patient was transferred to our hospital on 4 September 2025, for planned left iliac artery stent placement. Routine preoperative testing revealed unexpected cardiac abnormalities: troponin I elevation (1.052 μg/L; normal <0.04 μg/L), elevated B-type natriuretic peptide (1723.5 pg/mL; normal <100 pg/mL), and ECG showed ST-elevation in inferior (II, III, AVF) and anterior (V3–V6) leads, and with abnormal Q waves in the inferior leads. The patient was urgently transferred to cardiology, and vascular intervention was cancelled.

Coronary angiography showed normal coronary anatomy with TIMI grade 3 flow, excluding acute coronary syndrome.

**Figure 1:**
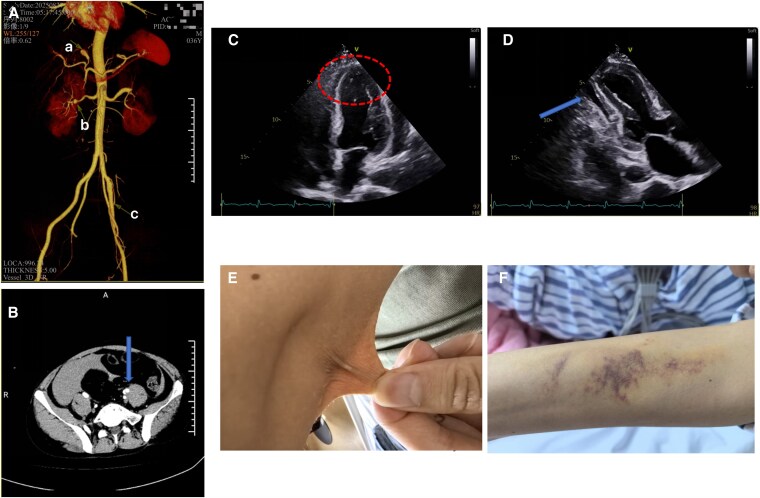
(*A*) Three-dimensional CT angiography (CTA) reconstruction demonstrating multiple vascular lesions in a patient with vascular Ehlers-Danlos syndrome (vEDS). The image shows: (*A*) hepatic artery aneurysm, (*B*) renal artery dissection, and (*C*) external iliac artery dissection. (*B*) Axial CT image showing left external iliac artery dissection (arrow) with visible intimal flap and false lumen formation. (*C*) Echocardiography shows mild-moderate apical ballooning (circled area). (*D*) Pericardial effusion (arrow) and lateral wall desynchrony. (*E*) A 36-year-old man demonstrating skin hyperextensibility upon manual traction. (*F*) This picture showed spontaneous bruising on the left forearm.

Echocardiography demonstrated left ventricular wall thickening, dyssynchronous contraction, mild-moderate apical ballooning, and pericardial effusion, consistent with stress cardiomyopathy (*Figure 1*), (Supplementary Video S1).

Acute myocarditis was considered in the differential diagnosis but was clinically ruled out based on the absence of viral prodrome, fever, and normal inflammatory markers at admission, and the typical wall motion pattern of stress cardiomyopathy.

A young male patient with multisystem arterial dissections and unexplained stress cardiomyopathy prompted a formal multidisciplinary team consultation. Genetics evaluation revealed characteristic vEDS features: thin lips, small earlobes, midface hypoplasia, skin hyperextensibility, and spontaneous bruising. Importantly, the patient had a previous spontaneous pneumothorax, an established early warning sign of vEDS.^4^ Family history showed a pattern of early cardiovascular deaths: his father and two paternal uncles died suddenly before age 50. Whole exome sequencing confirmed the presence of a heterozygous pathogenic variant in the COL3A1 gene (c.2284-2A>G), establishing a diagnosis of vascular EDS.

All invasive vascular procedures were permanently suspended due to prohibitive bleeding risks. Conservative management included blood pressure control with valsartan 40 mg daily and heart rate control with metoprolol extended-release 95 mg twice daily plus ivabradine 5 mg twice daily. Anticoagulation was avoided due to bleeding risk.

Follow-up troponin I showed decline (1.052→0.613→0.303 μg/L over 4 days) and echocardiography demonstrated improvement in stress cardiomyopathy. The patient was discharged with comprehensive long-term cardiovascular monitoring and family genetic counselling.

## Discussion

This case represents the first reported association between genetically confirmed vEDS and stress cardiomyopathy, providing new insights into the cardiac manifestations of type III collagen disorders. The stress cardiomyopathy observed in this patient met all diagnostic criteria established by the European Society of Cardiology.^5^ The combination of acute reversible left ventricular dysfunction, wall motion abnormalities extending beyond a single coronary territory, new electrocardiographic abnormalities, elevated cardiac biomarkers with disproportionately high BNP relative to troponin, a stressful trigger, and normal coronary angiography supported the diagnosis. This observation raises the question of whether vEDS confers an underlying predisposition to this condition.

### Proposed molecular mechanisms

Inflammatory state and TGF-β dysregulation in vEDS. Studies have demonstrated that vEDS patients exhibit significantly elevated plasma levels of TGF-β1 and TGF-β2, accompanied by upregulation of inflammatory markers including monocyte chemoattractant protein-1 and C-reactive protein.^6^ This provides direct evidence of systemic inflammatory activation in vEDS patients. Importantly, while these studies noted ‘no differences in downstream canonical TGF-β signalling pathways,’ the elevation may primarily reflect ‘chronic vascular injury’ rather than active pathological signalling, suggesting a state of chronic low-grade inflammation that could predispose to cardiac complications.

Type III collagen and myocardial function: vEDS manifestations originate from type III collagen defects. In the heart, type III collagen (11% of total) provides elasticity and participates in mechanical force transmission, while type I collagen (85%) provides tensile strength.^7^ This balance maintains normal myocardial compliance. Studies in dilated cardiomyopathy have shown that myocardial fibrosis is characterized by increased type I collagen accumulation with relatively decreased type III collagen deposition, fundamentally altering their ratio.^8^ Animal studies demonstrate that cardiac overexpression of type III collagen improves cardiac function by restoring collagen distribution balance.^9^ In vEDS, the primary defect in type III collagen production may be predisposed to pathological cardiac remodelling, whereas the relative excess of type I collagen could impair myocardial compliance and stress adaptation.

Proposed mechanistic model: Based on current evidence, we propose a multi-step mechanistic model for stress cardiomyopathy in vEDS: baseline vulnerability due to altered cardiac collagen I/III ratio and chronic inflammatory state;^1^ acute catecholamine surge triggered by severe vascular events, which is the classic pathogenic mechanism of stress cardiomyopathy;^2^ impaired myocardial stress adaptation due to defective type III collagen matrix;^3^ resultant acute cardiac dysfunction manifesting as stress cardiomyopathy.

### Clinical implications

This novel association has potential implications for vEDS management. Current vEDS guidelines focus primarily on vascular complications and do not specifically address cardiac monitoring protocols. Based on this case, we propose enhanced cardiac surveillance for vEDS patients, particularly during acute vascular events. We recommend: Baseline echocardiographic evaluation for all newly diagnosed vEDS patients;^1^ serial cardiac biomarker monitoring during acute vascular events;^2^ and conservative management approach for cardiac complications to avoid invasive procedures that could precipitate vascular catastrophe.

### Future research directions

This case opens several important research avenues: systematic cardiac assessment in vEDS cohorts to determine the prevalence of subclinical cardiac dysfunction;^1^ molecular studies examining type III collagen's role in myocardial stress response and the impact of altered collagen I/III ratios on cardiac function;^2^ investigation of TGF-β signalling pathways and inflammatory markers as potential biomarkers for cardiac risk stratification in vEDS patients;^3^ and development of cardiac monitoring protocols specifically tailored for vEDS patients. The identification of this novel association also suggests that other connective tissue disorders involving type III collagen may similarly predispose to stress cardiomyopathy, warranting broader investigation across the spectrum of collagen-related disorders. Ultimately, whether this represents a novel cardiac phenotype in vEDS-related to inflammation, collagen deficiency, and catecholamine susceptibility—requires further validation through additional cases and mechanistic studies.

### Limitations

Cardiac magnetic resonance (CMR) was not performed. CMR is the noninvasive gold standard for diagnosing MINOCA^10^ and can help differentiate stress cardiomyopathy from other myocardial diseases, including spontaneous coronary artery dissection (SCAD) and acute myocarditis. Therefore, while the clinical, electrocardiographic, angiographic, and echocardiographic findings strongly support stress cardiomyopathy, the absence of CMR limits definitive exclusion of alternative diagnoses such as SCAD or myocarditis. Our findings should be interpreted with this limitation in mind.

## Conclusions

We report the first case of stress cardiomyopathy in genetically confirmed vEDS, suggesting that *COL3A1*-related disorders may present with a previously unrecognized cardiac manifestation. The underlying mechanisms may involve chronic inflammatory activation, altered cardiac collagen I/III ratios, and impaired myocardial stress adaptation due to defective type III collagen matrix. This case provides a foundation for future research investigating cardiac manifestations in vEDS and highlights the importance of systematic cardiac evaluation and monitoring strategies tailored to this patient population. Recognition of this novel association may have broader implications for other type III collagen-related conditions.

## Patient perspective

Notably, the patient himself is a physician at a primary care hospital who was previously unaware of his own vEDS diagnosis. He states: ‘My experience underscores our limited exposure to rare diseases. I consent to share my case to enhance understanding of this condition among frontline clinicians.’

## Supplementary Material

xvag100_Supplementary_Data

## Declarations

### Data availability

No data were generated or analysed for this manuscript.
