# Supplementary material for: Stress cardiomyopathy in vascular Ehlers-Danlos syndrome: first case report and proposed mechanisms
Source: ESC Heart Fail. 2026 May 12;13(3):xvag100. doi: 10.1093/eschf/xvag100 (PMC13165414; doi:10.1093/eschf/xvag100)
Supplement: xvag100_Supplementary_Data [file xvag100_supplementary_data.zip › Patient Informed Consent Form.pdf]

患者知情同意书（Patient Informed Consent Form）

致患者：（To the Patient）

感谢您参与本次医疗病例的报道。为了促进医学科学的发展和医学教育，我们希望能将您的病例资料用于学术发表。请您仔细阅读以下内容，并在完全理解后自愿签署。

(Thank you for your participation in this medical case report. To promote medical science and education, we would like to use your case details for academic publication. Please read the following information carefully and sign voluntarily after full understanding.)

---

1. 病例信息（Case Information）

病例标题（Title of Case): Stress Cardiomyopathy in Vascular Ehlers–Danlos Syndrome: First Case Report and Proposed Mechanisms
（应激性心肌病在血管型埃勒斯-当洛斯综合征中的首次病例报告及机制探讨）

拟投稿期刊（Journal for Submission): ESC Heart Failure

2. 同意内容（Consent Provisions）

本人已阅读并理解上述信息，并在此自愿同意（I have read and understood the above information and hereby voluntarily consent to):

[√] 发表我的匿名病例细节，包括病史、症状、体征、实验室检查、影像学结果、诊断、治疗及预后。
(The publication of my anonymized case details, including medical history, symptoms, signs, laboratory tests, imaging findings, diagnosis, treatment, and outcome.)

[√] 发表我的匿名影像学图片（如心电图、超声心动图、CT 等），这些图片可能会被用于印刷和在线出版。
(The publication of my anonymized medical images (e.g., ECG, Echocardiogram, CT scans), which may be used in both print and online publications.)

[√] 发表我的匿名遗传学数据（如 COL3A1 基因变异信息）。
(The publication of my anonymized genetic data (e.g., COL3A1 gene variant information).)

[√] 我理解并同意，尽管已采取匿名化措施，但无法完全排除根据病例细节被识别的可能性。

(I understand and agree that, despite all efforts to maintain anonymity, there is a small possibility that I could be identified based on the case details.)

3. 患者权利（Patient's Rights）

我理解我的参与是自愿的。
(I understand that my participation is voluntary.)

我理解我有权随时撤回同意，无需提供理由。但撤回同意不会影响在撤回前基于本同意书已进行的发表活动。
(I understand that I have the right to withdraw this consent at any time without giving a reason. However, such withdrawal will not affect any actions of publication that have already been taken based on this consent prior to the withdrawal.)

我理解我不会从本次发表中获得任何经济报酬。
(I understand that I will not receive any financial compensation for this publication.)

患者声明（Patient's Statement）

本人确认，已获得足够的时间进行考虑并提出问题，且所有问题均已得到满意的解答。本人完全理解本同意书的内容，并自愿同意上述所有条款。

(I confirm that I have been given sufficient time to consider and ask questions, and all my questions have been answered to my satisfaction. I fully understand the contents of this consent form and voluntarily agree to all the provisions above.)

患者签名（Patient's Signature): 陈令新
患者姓名正楷（Printed Name): ________________________
日期（Date): ______2025 年 11 月 20 号__________________

医生/研究者签名（Physician/Investigator Signature): [illegible]
医生/研究者姓名正楷（Printed Name): ________________________
日期（Date): ____2025 年 11 月 20 号_____________________
