# Supplementary figures and images for: Stress cardiomyopathy in vascular Ehlers-Danlos syndrome: first case report and proposed mechanisms
Source: ESC Heart Fail. 2026 May 12;13(3):xvag100. doi: 10.1093/eschf/xvag100 (PMC13165414; doi:10.1093/eschf/xvag100)

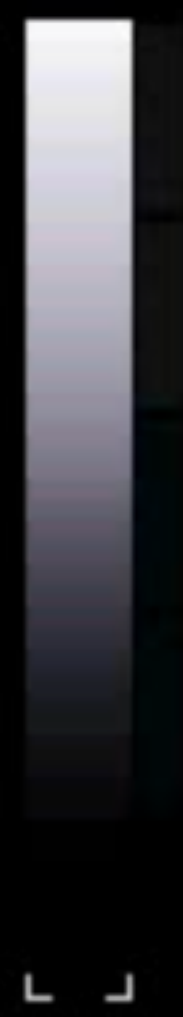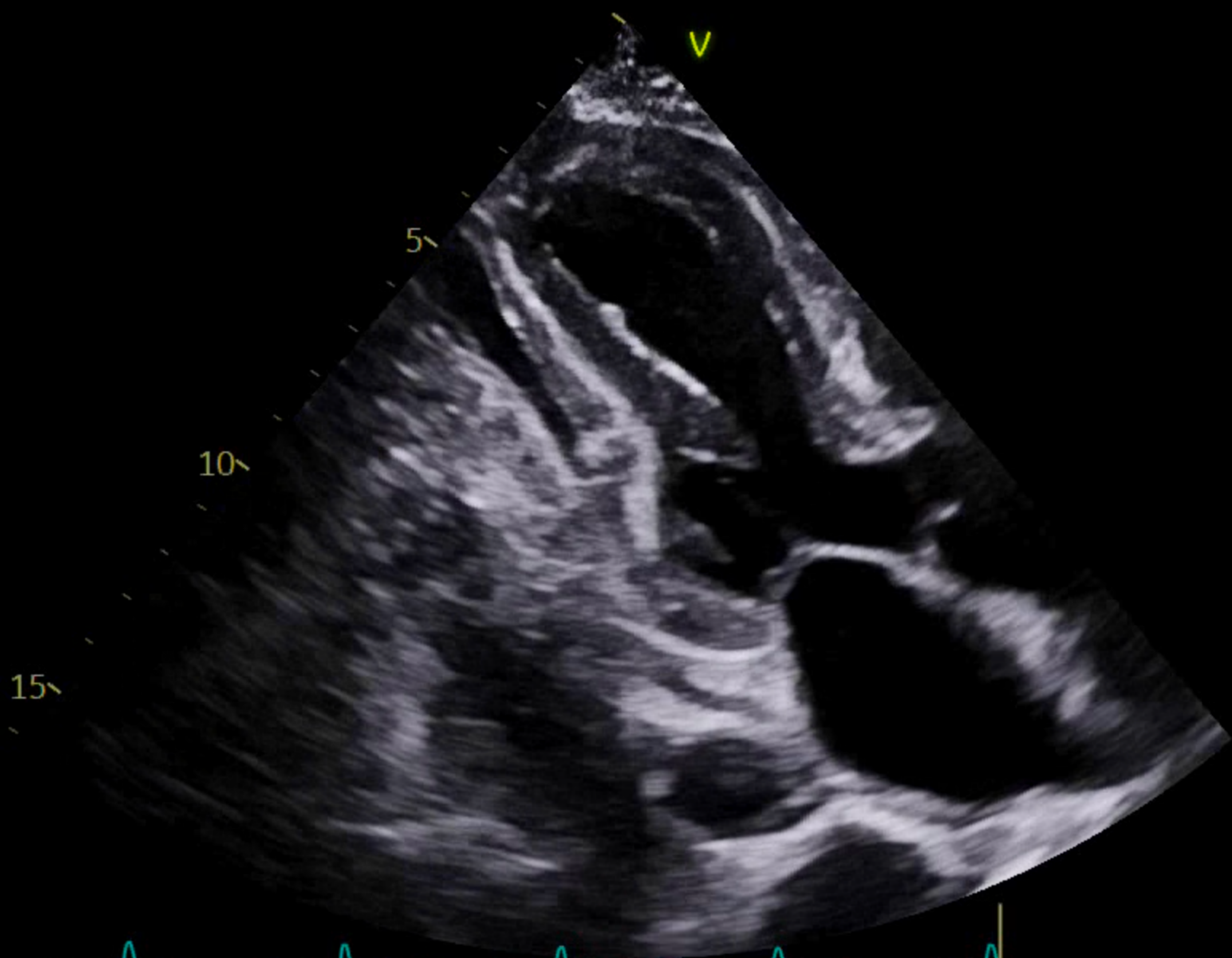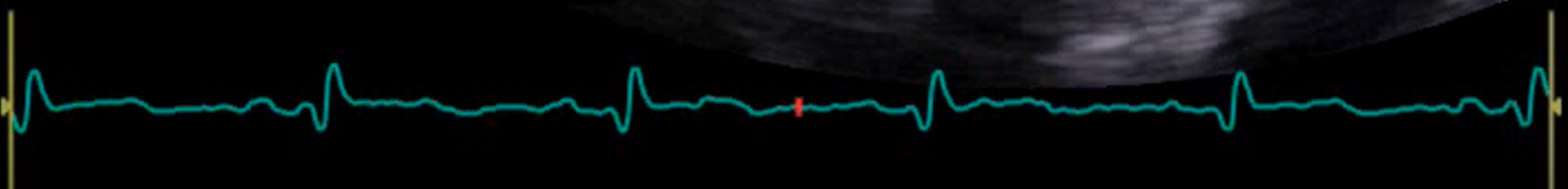

Supplement: xvag100_Supplementary_Data [file xvag100_supplementary_data.zip › ideo 1 still image 2.pdf]

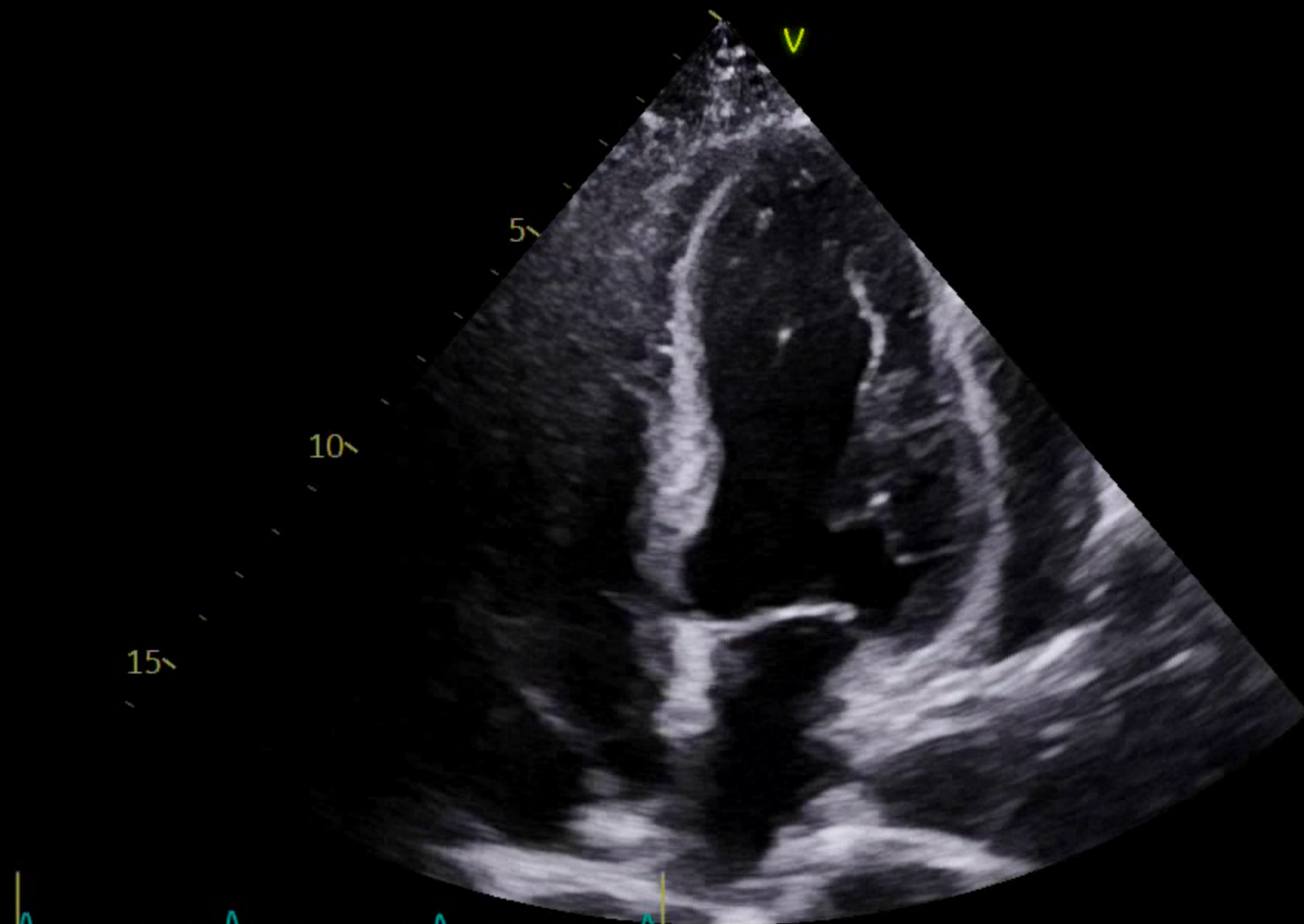

Supplement: xvag100_Supplementary_Data [file xvag100_supplementary_data.zip › Video 1 still image 1.pdf]
